# Effects of Semaglutide Treatment on Psoriatic Lesions in Obese Patients with Type 2 Diabetes Mellitus: An Open-Label, Randomized Clinical Trial

**DOI:** 10.3390/biom15010046

**Published:** 2025-01-01

**Authors:** Jelena Petković-Dabić, Ivana Binić, Bojana Carić, Ljiljana Božić, Sanja Umičević-Šipka, Nataša Bednarčuk, Saša Dabić, Mirna Šitum, Snježana Popović-Pejičić, Miloš P. Stojiljković, Ranko Škrbić

**Affiliations:** 1 Center for Biomedical Research, Faculty of Medicine, University of Banja Luka, 78000 Banja Luka, Bosnia and Herzegovina; jelena.petkovic@kc-bl.com (J.P.-D.); bojana.caric@kc-bl.com (B.C.); sanja.umicevic@kc-bl.com (S.U.-Š.); natasa.bednarcuk@med.unibl.org (N.B.); snjezana.popovic-pejicic@med.unibl.org (S.P.-P.); milos.stojiljkovic@med.unibl.org (M.P.S.); 2 Clinic of Skin and Venereal Diseases, University Clinical Centre of the Republic of Srpska, 78000 Banja Luka, Bosnia and Herzegovina; 3 Faculty of Medicine, University of Niš, 18000 Niš, Serbia; ivanabinic@gmail.com; 4 Department of Internal Medicine, Faculty of Medicine, University of Banja Luka, 78000 Banja Luka, Bosnia and Herzegovina; 5 Department of Microbiology and Immunology, Faculty of Medicine, University of Banja Luka, 78000 Banja Luka, Bosnia and Herzegovina; ljiljana.bozic@med.unibl.org; 6 Faculty of Medicine Foča, University of East Sarajevo, 71123 Istočno Sarajevo, Bosnia and Herzegovina; sajodab@gmail.com; 7 Department of Dermatology and Venerology, University Hospital Center “Sestre Milosrdnice”, University of Zagreb, 10000 Zagreb, Croatia; mirna.situm@kbcsm.hr; 8 Croatian Academy of Science and Arts, 10000 Zagreb, Croatia; 9 Academy of Science and Arts of the Republic of Srpska, 78000 Banja Luka, Bosnia and Herzegovina; 10 Department of Pharmacology, Toxicology and Clinical Pharmacology, Faculty of Medicine, University of Banja Luka, 78000 Banja Luka, Bosnia and Herzegovina; 11 Department of Pathologic Physiology, First Moscow State Medical University I.M. Sechenov, 119435 Moscow, Russia

**Keywords:** psoriasis, T2DM, semaglutide, PASI, DLQI, cytokine

## Abstract

Psoriasis is a chronic inflammatory skin disease with relapsing nature. Estimates are that approximately 2–3% of the world’s population suffers from this disease. More severe forms of psoriasis are conditions of high inflammation, which is confirmed by the clinical picture and numerous inflammatory parameters such as C-reactive protein (CRP), cytokines and homocysteine, which vary with disease activity. The objective of this clinical study was to investigate the effect of GLP-1 receptor agonist semaglutide therapy on pro-inflammatory factors in the serum and the severity of the clinical picture of psoriasis in obese patients with type 2 diabetes mellitus (T2DM) on chronic metformin therapy. This randomized clinical study was conducted on 31 psoriatic patients with T2DM that were randomized into two groups: one that received semaglutide during the 12-week trial (*n* = 15), while the second was control (*n* = 16). The results demonstrated that the severity of the clinical picture of psoriasis, determined by the Psoriasis Area and Severity Index (PASI) score, was significantly better after the administration of semaglutide (the median baseline PASI score in patients treated with semaglutide was 21 (IQR = 19.8), while after 12 weeks of therapy the score was 10 (IQR = 6; *p* = 0.002). Also, the quality of life in the group of patients who received the drug, measured by the Dermatology Life Quality Index (DLQI), improved significantly after 3 months (a median baseline DLQI score in the semaglutide group was 14 (IQR = 5) at the beginning of the study, and after 12 weeks of treatment the median DLQI score was 4 (IQR = 4; *p* = 0.002)). The use of semaglutide led to a significant decrease in pro-inflammatory cytokines in the serum (IL6), as well as a significant decrease in CRP values (*p* < 0.05). A significant decrease in the body mass index (BMI) value in the semaglutide-treated group was also identified, as well as a significant decrease in the level of low-density cholesterol (LDL) (*p* < 0.05). In conclusion, semaglutide, based on its systemic anti-inflammatory characteristics, could contribute to the treatment of psoriatic obese patients with T2DM.

## 1. Introduction

Psoriasis is a chronic inflammatory skin disease with relapsing nature. It has been estimated that 2–3% of the world’s population suffers from this disease [1]. The most common type of psoriasis is plaque psoriasis characterized by dry, itchy, raised skin patches (plaques) covered with scales. Histopathological findings of the plaques reveal the extensive hyperproliferation of epidermal keratinocytes and excessive inflammatory cell infiltration including neutrophils, dendritic cells, and T lymphocytes [2,3]. Although the pathogenesis of psoriasis is not clear, a large amount of data indicates a complex interaction between epidermal keratinocytes and inflammatory cells. More severe forms of psoriasis are associated with a high degree of inflammation, that are confirmed by psoriatic skin lesions and numerous inflammatory mediators such as TNFα, IL-1, IL- 6, IL-8, IL-17, IL-22, IL-23, INF-ɣ, homocysteine and hsCRP, which highly correspond with disease activity [4]. Increased levels of IL-6 were observed, not only in psoriatic lesions and keratinocytes, but also in the plasma/serum of patients with psoriasis, compared to the levels of IL-6 in healthy individuals. It is also shown that IL-6 affects the hyperproliferation of epidermal cells that is characteristic for psoriasis [5,6].

Besides its local manifestation, psoriasis is a multisystemic inflammatory disease that can affect different organs and organic systems and can be associated with various comorbidities such as dyslipidemia, hypertension, polyarthritis, insulin resistance and metabolic syndrome, diabetes mellitus, and cardiovascular diseases [7,8,9]. The prevalence of Type 2 Diabetes Mellitus (T2DM) in patients with psoriasis ranges from 4.4 to 54% and is higher in patients with moderate to severe psoriasis compared with the mild form of the disease [10]. Although the exact mechanism linking T2DM and psoriasis is still unclear, it is assumed that chronic inflammation may play a central role in both cases [11,12,13].

Recently, the glucagon-like peptide 1 receptor agonists (GLP-1 RA) have been extensively studied as a possible treatment for T2DM, and GLP-1 RA together with dipeptidyl peptidase-4 (DPP-4) inhibitors are now in widespread clinical use in these patients [14]. Data from various studies suggest that GLP-1 RA therapy achieves anti-inflammatory effects on the liver and vascular system, including endothelial cells of the aorta and veins, brain, kidneys, lungs, testicles and skin, by reducing the production of inflammatory cytokines and the infiltration of immune cells [15,16,17]. The anti-inflammatory and anti-oxidative effects of GLP-1 RA have been confirmed in animal studies, in which the reduction of pro-inflammatory cytokines TNF-a and IL-1β, as well as the oxidative stress markers, was shown, in addition to the effect on glycemic control and reduction of Body Mass Index (BMI), which also contributes to the reduction of inflammation [18,19,20].

GLP-1 RA may be useful for the treatment of chronic inflammatory diseases including nonalcoholic steatohepatitis, atherosclerosis, neurodegenerative disorders, diabetic nephropathy, asthma, and psoriasis [21,22,23,24]. Semaglutide is an improved, highly potent GLP-1 RA that is protected from DPP-4 cleavage and further optimized for high-affinity albumin binding, allowing once-weekly administration [16]. There are insufficient data in the literature on the effects of semaglutide on the outcomes of psoriasis treatment. One of the observed anti-inflammatory effects of semaglutide is the effect on the reduction of CRP, which can be used as a parameter of the disease course and the psoriasis prognosis, but additional research is necessary to fully clarify this mechanism [14,15,16,17]. In a case study, the effect of semaglutide was evaluated in a 73-year-old psoriatic patient with diabetes mellitus. It was found that after 4 months of semaglutide therapy, glycemic parameters improved, the patient’s body weight decreased, the Psoriasis Area Severity Index (PASI) score decreased by 19% compared to baseline, and the quality of life, assessed by Dermatology Life Quality Index (DLQI), improved significantly [21].

Based on the literature review, there are currently no clinical trials that have investigated the effect of semaglutide on the inflammatory response and course of psoriasis in obese patients with T2DM. Therefore, the aim of this clinical study was to examine the effects of 12-week semaglutide treatment in obese psoriatic patients with T2DM on lipid status, glycemic control, inflammatory markers, and clinical course of plaque lesions associated with quality of life.

## 2. Materials and Methods

### 2.1. Ethics

The study was conducted at the Clinic of Skin and Venereal Diseases of the University Clinical Centre of the Republic of Srpska (UCC), Banja Luka, Bosnia and Herzegovina. The study was performed in line with the principles of the Declaration of Helsinki (revised version from 1983), and in accordance with the rules of the Ethics Committee for Research on Humans and Biological Material of the Faculty of Medicine, University of Banja Luka, the Republic of Srpska, Bosnia and Herzegovina, as well as the Ethics Committee of the UCC (Decision number: 01-19-40-2/22). Each participant also received written information with details related to the study. Informed consent was signed in written form by each participant before any study procedure. This trial has been registered at http://www.clinicaltrials.gov/ as NTC06475586 (accessed on 1 July 2024) .

### 2.2. Participants and Study Groups

#### 2.2.1. Participants

A total of 31 patients of both sexes, 18–70 years of age, were divided into two groups: (a) treatment group of patients with psoriasis and T2DM who received the drug semaglutide along with standard metformin antidiabetic therapy (*n* = 15), and (b) control group of patients with psoriasis and T2DM, but who remained on the current metformin therapy and did not receive the semaglutide (*n* = 16). The patients were following a diet for diabetes and obesity recommended by nutritionists. No different approach to diet plan was recommended during the study. For those patients who had high LDL cholesterol, the introduction of lipid lowering therapy was postponed due to possible impact of statins on interleukin levels. For all patients, the diagnosis of psoriasis was made on the basis of a typical clinical feature, the existence of clearly defined erythematous plaques with non-adherent whitish squamous, determined by clinical examination and PASI scoring.

#### 2.2.2. Inclusion Criteria

Patients who signed an informed consent to participate in the study, with a typical clinical feature of moderately-severe to severe plaque psoriasis (PASI SCORE ≥ 10) and T2DM diagnosed at least 6 months before inclusion in the study and treated with metformin as a therapy, were included in the study.

#### 2.2.3. Non-Inclusion Criteria

The patients with other forms of psoriasis, other chronic inflammatory diseases, or those taking drugs that can cause the worsening of psoriasis such as lithium, systemic antimalarial drugs, or systemic corticosteroids for at least 3 month, systemic therapy of vulgaris psoriasis 3 months before inclusion in the study, and patients on therapy with other GLP-1 RA except semaglutide (liraglutide, dulaglutide, lixisenatide), SGLT-2 inhibitors (empagliflozin and dapagliflozin) and NSAIDs, photo UVB therapy, and patients with medullary thyroid cancer in their family history. Patients who did not personally sign consent to participate in the study were not included in the study.

#### 2.2.4. Criteria for Exclusion from the Study

Patients requesting for exclusion from the study, patients diagnosed with another pathological condition, adverse reactions to semaglutide described in the available Patient information leaflet (PIL): nausea, vomiting, allergic reaction at the localization of drug administration, were excluded from the study.

#### 2.2.5. Study Design

This was an open labeled interventional cohort study with two groups of participants.

#### 2.2.6. Treatment Group

Patients with T2DM and psoriasis (*n* = 15), who were on standard metformin therapy in the maximally tolerated dose, were assigned to receive semaglutide in standard treatment doses for the period of 12 weeks. The semaglutide therapy was initiated according to standard treatment guidelines for overweight patients with T2DM, with BMI > 30 kg/m^2^, and HbA1c > 7% with a dose of 1.0 mg/patient/week, during the period of 12 weeks. The same cut-off values of BMI and HgbA1C were applied for controls.

The patients treated with semaglutide received the drug free of charge, and upon the recommendation of an endocrinologist.

#### 2.2.7. Control Group

This group consisted of T2DM patients with psoriasis, who were already on metformin therapy in the maximally tolerated dose but were not assigned to receive any of GLP-1 receptor agonists.

During the 12 weeks of the study, the patients were contacted by phone, every month—a total of 3 times—in order to confirm the administration of the drug or the possible existence of adverse reactions to the drug, as described in the PIL. Both groups were allowed to take the topical keratolytic therapy of salicylic acid.

### 2.3. Clinical and Demographic Characteristics of Patients

All demographic characteristics of the patients, as well as anthropometric parameters, were also collected. Medical history was used for general demographic and social epidemiological data (date of birth, age, gender), as well as anthropometric measurements (body height and weight, and BMI). To assess the disease activity, i.e., skin surface affected by changes (erythema, infiltration and extent of squamous matter), the PASI score was used. The assessment of the clinical feature of psoriasis using the PASI score, as well as the Dermatology Life Quality Index (DLQI) were performed two times, at the beginning and at the end of the study.

### 2.4. Biochemical Analyses

Patients’ blood samples for biochemical analyses were collected 12–14 h after the last meal, two times during the study, at the beginning and at the end of the study. The concentrations of fasting glucose, fasting insulin, HgbA1C, CRP, homocysteine, total cholesterol, triglycerides, LDL and HDL cholesterol were assessed. These analyses were done at the Institute for Laboratory Diagnostics of the UCC.

### 2.5. Cytokine Quantification

The serum levels of IL-1β, IL-6, IL-17 and IL-23 were measured by means of the Enzyme Linked Immunosorbent Assay (ELISA) technique with commercial diagnostic kits BioLegend ELISA MAX Deluxe Sets (BioLegend, San Diego, CA, USA) and at the Center for Biomedical Research of the Faculty of Medicine of the University of Banja Luka.

### 2.6. Statistical Analysis

R version 4.2.3 was used for statistical analysis. Descriptive statistics were used to summarize or describe the central tendency of the data sets and their dispersion. For the analysis of numerical data, the skewness and kurtosis values as well as a Shapiro–Wilk test were used. The results were also shown graphically, such as charts if data were normally distributed, and box whisker plots if data were not normally distributed. Paired *t*-test was used for the comparison of mean values between two matched samples. In the event of deviations from the normal data distribution, the Wilcoxon signed-rank test was used. For comparison of the differences between two independent groups when the distribution of the variables was not normal, the Mann-Whitney U test was used. The Pearson’s χ^2^ test was used to compare the frequency of occurrence of the analyzed categorical variables when observing one or more samples. Statistical hypotheses were tested at the significance level (α) of *p* ≤ 0.05 and *p* < 0.001.

## 3. Results

A total of 31 participants (mean ± SD age: 57.97 ± 10.74), six (19.35%) women and 25 (80.65%) men were included in the study. There was no significant difference between the two groups for age and sex distribution, alcohol consumption and smoking status, and comorbidities, nor for BMI, PASI score values, DLQI score values, total glucose, cholesterol, and HbA1C at baseline (Table 1). During the observed period, three patients were excluded from the study; two of them due to drug side effects (nausea, vomiting), and one patient due to exacerbation of the disease.

**Figure 1:**
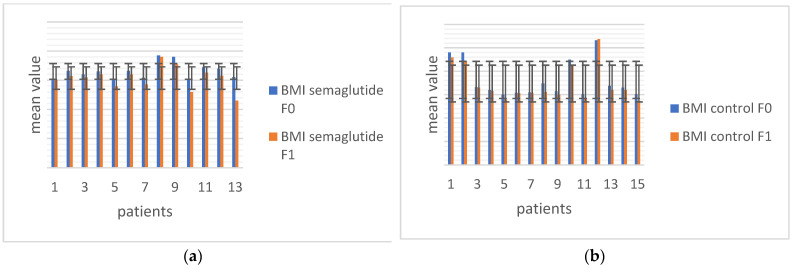
The mean value and error bars of standard deviations of BMI at baseline (F0) and after 12 weeks (F1). (**a**) Patients treated with semaglutide, (**b**) control group.

The mean value of BMI in all patients treated with semaglutide was significantly decreased after 12 weeks (from 33.04 ± 2.7 to 30.7 ± 3.8; *p* = 0.001). However, a significant decrease in BMI value was also observed in the control group, in which the mean value of BMI before therapy was 36.3 ± 7.9, and after 12 weeks it was 34.8 ± 7.9 (*p* < 0.001); (Figure 1).

A significant decrease of LDL was observed in group of patients treated with semaglutide (*p* < 0.05), but not in control group of patients (*p* > 0.05). A significant decrease of HgbA1C was observed in both groups of patients (*p* < 0.05, *p* < 0.001). There was a slight decrease in glucose level in the semaglutide group but this decrease was not significant (Table 2).

Although a decrease in inflammatory parameters, CRP, IL-1β, IL-6, and IL-23 were observed in both groups of patients, it was statistically significant only for CRP and IL-6 in the group treated with semaglutide (*p* < 0.05). Interleukin-17 was not detected in the serum of patients in the present study (Table 3).

Further, we compared the baseline values of CRP and IL-23 between the semaglutide group and the control group. Statistical analysis showed that there was a significant difference in the median values of IL-23 between semaglutide and control groups (Table 4).

**Figure 2:**
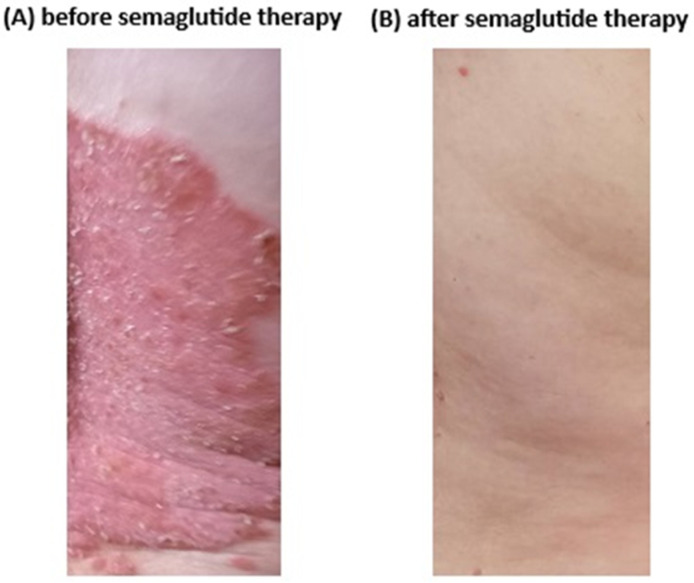
The skin of the patient’s right lumbosacral area treated with semaglutide with a good responding profile; (**A**) at baseline, and (**B**) after 12 weeks of treatment.

**Figure 3:**
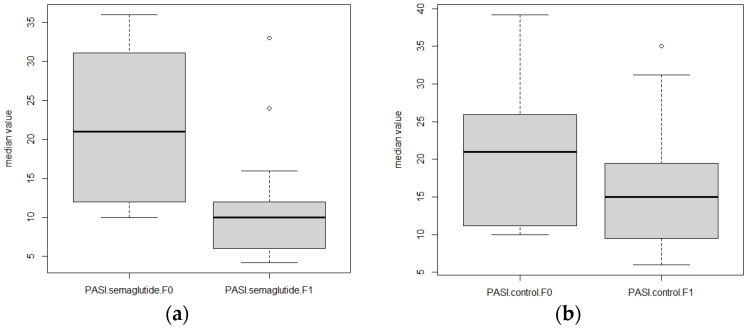
Box plot of median PASI score values at baseline (F0) and after 12 weeks (F1); (**a**) patients treated with semaglutide, (**b**) control group.

**Figure 4:**
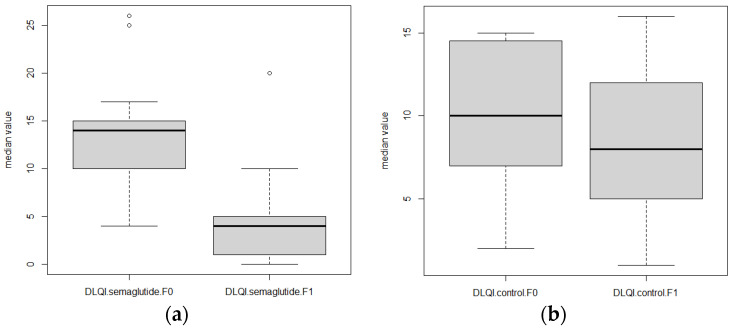
Box plot of median DLQI score values at baseline (F0) and after 12 weeks (F1) in patients treated with semaglutide (**a**) and control group (**b**).

The treatment with semaglutide was very effective in reducing the psoriatic lesions, as is shown in Figure 2. The median baseline PASI score in patients treated with semaglutide was 21 (IQR = 19.8), while after 12 weeks of therapy the score was 10 (IQR = 6; *p* = 0.002; Figure 3). In the control group, the mean baseline PASI score was 20.6 (IQR = 8.9), and after 12 weeks it was 15.9 (IQR = 8.7; *p* = 0.03; Figure 3). Furthermore, a median baseline DLQI score in the semaglutide group was 14 (IQR = 5) at the beginning of the study, and after 12 weeks of treatment the median of the DLQI score was 4 (IQR = 4; *p* = 0.002). However, the mean DLQI score in the control group was also decreased after 12 weeks (10.1 [IQR = 4.3] versus 8.1 [IQR = 4.8]; *p* = 0.007), but this decrease was much lower compared to the treated group (Figure 4).

The statistical differences in psoriasis severity at baseline and after 12 weeks of treatment are summarized in Table 4. It was found that there was a significant improvement in the clinical outcome in patients treated with semaglutide. However, in the control group there was no difference in psoriasis severity at the baseline compared to the end of the study (Table 5).

At 12 weeks, six out of 13 (46%) patients achieved PASI 90 and one out of 13 (8%) achieved PASI 100 response in the semaglutide group, while these numbers in the control group were only one out of 15 (7%) and none, respectively.

By analyzing different DLQI classes, it was observed that semaglutide significantly improved the quality-of-life score, while this was not observed in the control group (Table 6).

The DLQI score, marked as “a very large impact of psoriasis on the quality of life”, was positively answered by almost 90% of patients on average at the beginning of the study, while only 10% of them gave this answer after a three-month period of semaglutide use.

## 4. Discussion

The findings of this study showed a significant (52.4%) decrease of the PASI score after 12 weeks of semaglutide treatment in obese psoriatic patients with T2DM, compared to the control group of patients (22.8%). The results also demonstrated a significant improvement in the quality of life of psoriasis patients who received semaglutide during the investigated period. Until now, there have been no clinical trials studying the effects of semaglutide in obese psoriatic patients with T2DM. However, there were only two case-reports describing the effects of semaglutide in psoriatic patients with T2DM. Constanza et al. [21] showed a significant improvement of the clinical characteristics of psoriasis as indicated by the PASI score in one patient who also suffered from T2DM. They demonstrated a significant improvement in the PASI score by as much as 19%, as well as the significant improvement in the quality of life expressed through the DLQI, associated with a significant loss of body weight and improvements in glycemic parameters such as glycemia and HgbA1C. Another case report, published by Malavazos et al. [22], also confirmed a significant improvement in PASI and DLQI after a ten-month period of semaglutide administration, in an obese patient with psoriasis suffering from T2DM. The meta-analyses of clinical trials in which the GLP-1 RA liraglutide was used in psoriatic patients with T2DM demonstrated positive effects on PASI score and fasting plasma glucose level, but also showed that liraglutide treatment in these patients had no effects on DLQI, BMI, or HbA1c [23]. Similar to the results of case reports, we also demonstrated a significant improvement in PASI and quality of life, as well as the decrease in the value of HgbA1C, in the group of patients treated with semaglutide.

Obesity is a risk factor for the occurrence and worsening of existing psoriasis, and it is considered that reducing body weight in overweight people can improve the severity of symptoms of psoriasis [24]. The results of a randomized trial by Jensen et al. [25], which included patients suffering from both psoriasis and obesity, showed that the intervention group, which was subjected to a low-calorie index diet, had a greater loss of body weight, but also a significant decrease in PASI compared to the control group. It has even been proven that weight loss and improvement in PASI were maintained to a significant extent even after 12 months [26]. It has been shown that GLP-1 RAs treatment is associated with significant weight loss, along with lowering plasma glucose levels [23]. In the study performed by Milton Packer [27], it was proven that the activity of semaglutide directly affects the improvement of glycemic control, which leads to an effective loss of body weight, but also reduces the dysfunction of fatty tissue and inflammation, and at the same time improves the clinical characteristics of psoriasis. Similarly to these reports, a significant decrease in BMI in the group treated with semaglutide was observed in our study. This was associated with a slight, but not significant decrease in plasma glucose level which could be ascribed to good glycemic control obtained with metformin.

The results of our study also showed that LDL cholesterol level in patients who received semaglutide for a 3-month period was significantly lower than at the beginning of the study. The results of a meta-analysis from 2021 that included 76 placebo-controlled trials in which different GLP-1 RAs were used, and with a total number of 39,246 T2DM patients, clearly showed that GLP-1 RAs treatment for a period of at least 12 weeks significantly lowered HgbA1c, fasting glucose, and body weight. However, only semaglutide was effective in lowering the level of lipids, namely LDL and total cholesterol in the serum [28], which corresponds to the results of our study.

A review of the literature reveals an extraordinary interest in examining the pleiotropic effects of GLP-1 RA over the past few years, and various studies determined the anti-inflammatory effects of these drugs in humans, which have already been proven in a couple of preclinical models [29]. Some recent studies demonstrated that GLP-1 RAs may be useful for the treatment of chronic inflammatory diseases including nonalcoholic steatohepatitis, atherosclerosis, neurodegenerative disorders, diabetic nephropathy, asthma, and psoriasis [15,16,17]. New indications for GLP-1 RAs, apart from T2DM, such as neurodegenerative diseases, type 1 diabetes mellitus, and psoriasis, have been investigated as well [30]. It seems that our study is the first that investigated the effect of semaglutide on inflammatory response and clinical course of T2DM obese patients, suffering from vulgar plaque psoriasis.

One of the anti-inflammatory effects of semaglutide is the effect on the reduction of hsCRP, which can be used as a parameter for disease course and the prognosis of psoriasis. In the study by Verma et al. [31], the analysis of three randomized, double-blind, placebo-controlled clinical trials demonstrated a reduction of CRP in obese patients after 68 weeks of continuous semaglutide treatment. Similar to this, a significant decrease in CRP values was observed in diabetic patients after six months of semaglutide administration [32]. These results are in accordance with the findings of our study, where we demonstrated a statistically significant decrease in CRP values after 12 weeks of semaglutide administration in obese, psoriatic patients with T2DM.

It has already been found that liraglutide led to the decreased expression of IL-17 mRNA in psoriasis plaques [33]. The effectiveness of GLP-1R agonists is likely to be ascribed to the amelioration of adipose tissue dysfunction and minimizing the source of adipocytokines that can induce and promote inflammation in the skin [34]. The expression of GLP-1Rs was shown in human skin changes of plaque psoriasis, but not in human keratinocyte cultures, which further implies that the presence of these receptors in psoriatic plaques is a consequence of immune cell infiltration [35]. The results of a meta-analysis from 2013 by Dowlatshahi et al. [36] comparing serum inflammatory markers such as IL-1b, IL-6, IL-10, CRP, ICAM-1, E-selectin and TNF-α, in patients with psoriasis and healthy control groups, indicated modest but significantly elevated levels of pro-inflammatory cytokines in the serum of patients with moderately severe psoriasis. This is similar to the results of our study, where we confirmed the existence of elevated levels of pro-inflammatory cytokines in patients with moderately severe and severe psoriasis, especially of IL-6. After the 12-week treatment with semaglutide, the level of IL-6 decreased significantly, while the level of IL-6 in the control group was not changed. The decrease in pro-inflammatory cytokine levels further implies the anti-inflammatory effect of semaglutide, which was one of the aims of the present study. However, the values of IL-1b were not changed neither in the group receiving semaglutide, nor in the untreated group.

The role of IL-23/IL-17 inflammatory axes in psoriasis, as well as in various metabolic diseases, has been investigated intensively. The experimental study by Chen et al. [37] clearly showed the positive effect of liraglutide on the size of psoriatic plaques in obese mice, which was associated with inhibition of the IL 23/Th-17 pathway. However, unlike liraglutide, there are no available clinical studies on the effect of semaglutide administration on the levels of IL-17 and IL-23 in the serum of psoriatic obese patients with T2DM.

In our study, we did not show a significant decrease of IL-23 levels in the serum of patients after 12 weeks of semaglutide treatment, and the presence of IL-17 was not even detected. These data may point to the literature findings indicating a very low detectability of this pro-inflammatory cytokine in the serum of psoriatic patients, but also significantly higher values in the erythemo-squamous plaque itself, where this value was obtained by an immunohistochemical method from tissue samples [38]. The recently published meta-analysis did not show a significant presence of circulating cytokines in patients with psoriasis, and no significant correlation was demonstrated between the levels of IL-4, IL-12, IL-22, IL-23, IL-35, IL-36 and TGFβ with the severity of the clinical symptoms of psoriasis [39]. Similar results were obtained from the study by Yilmaz et al., where 70 patients suffering from various forms of psoriasis and 50 healthy participants (control group) were examined, and in which no statistical significance related to IL-17 was determined, neither in the patients nor in the control group [40]. In our previous clinical study on psoriatic patients, we observed that high doses of vitamin D supplementation led to a significant decrease in pro-inflammatory cytokines (IFN-7, TNF-α, IL-1β, IL-6, IL-8, and IL-17) and high-sensitivity C-reactive protein (hsCRP), whereas the production of anti-inflammatory cytokines (IL-10, IL-5) were up-regulated [3].

It should be noticed that psoriatic patients recruited in our study were patients with T2DM who were already on continuous metformin therapy. There are many experimental and clinical studies confirming the anti-inflammatory properties of metformin that could influence the level of cytokines [41] and we can speculate that these findings could be the consequence of continuous metformin treatment.

Further review of the literature shows that patients with psoriasis have a significantly higher level of homocysteine in the serum. It is assumed that homocysteine initiates the immunoinflammatory process by increasing the level of cytokines, then by activating Th1 and Th17 cells and neutrophils, with the suppression of T regulatory cells. Having that in mind, homocysteine could be one of the target molecules in the treatment of psoriasis [42]. The results of our previous study clearly showed that an increased homocysteine level in psoriatic patients was significantly attenuated following three months of high-dose vitamin D supplementation [3]. The results of a meta-analysis from 2019 [43] showed that patients with psoriasis had significantly higher homocysteine levels compared to healthy controls. On the other hand, the study published by Ataseven et al. [44], which examined the values of inflammatory markers in serum of patients with psoriasis, showed that homocysteine values were not different from healthy controls. In our study, we found higher values of homocysteine concentration in obese patients with T2DM, but with no significant difference between the treated and untreated groups. However, this is not surprising, bearing in mind that long-term metformin therapy could elevate the homocysteine concentration in these patients [45].

One of the limitations of our study is the limited number of patients treated with semaglutide, which is also a possible reason for not finding statistical significance in the levels of certain inflammatory markers at the end of the study. Some further double-blind, placebo-controlled studies with a larger number of psoriasis patients could contribute to a better understanding of the role of semaglutide in blocking the inflammatory response in psoriasis. Besides, psoriasis and T2DM are both associated with increased cardiovascular risk, and while semaglutide has been shown to improve lipid profiles and reduce BMI in this study, the cardiovascular outcomes were not registered, as it was assumed that the observational period was too short.

Although metformin is known to exert anti-inflammatory effects that can influence the levels of cytokines, this notion did not influence the effects of semaglutide, since patients in both semaglutide and control groups were treated with the same doses of metformin. As a result, all the differences in parameters monitored were ascribed to semaglutide only.

Limitations:

1. Sample size: The study included only 31 participants, which may limit the generalizability of the findings to a broader population.
2. Open-label design: Both the patients and the researchers were aware of the treatments being administered, therefore introducing potential for bias, as participants might have had heightened expectations for effectiveness of semaglutide, influencing their self-reported quality-of-life improvements.
3. Limited duration of follow-up: The follow-up period in this study was only 12 weeks, which may not be sufficient to observe long-term effects or potential side effects of semaglutide on psoriasis and T2DM.
4. Cytokines: The study measured the levels of several pro-inflammatory cytokines, including IL-1β, IL-6, IL-23, and CRP, as indicators of inflammation in psoriasis. While a significant reduction in IL-6 and CRP was observed in the semaglutide group, IL-1β and IL-23 did not show significant changes, and IL-17 was not detected at all. This inconsistency raises questions about the mechanisms by which semaglutide exerts its anti-inflammatory effects.
5. Exclusion of other psoriasis treatments: The study excluded patients who were using other GLP-1 receptor agonists, corticosteroids, or other systemic therapies that could affect psoriasis. While this was necessary to isolate the effects of semaglutide, it also means that the results cannot be generalized to patients who are receiving combination therapies, which are common in clinical practice.

## 5. Conclusions

This is the first clinical trial that has studied the effects of 12-week semaglutide treatment in obese psoriatic patients with T2DM which showed multiple clinical and scientific outcomes. Firstly, the results of this study clearly confirmed the class effect of GLP-1 RA in the treatment of psoriasis. Secondly, besides its evident effects on weight control, semaglutide has proven pleiotropic effects on CRP and proinflammatory cytokines. Thirdly, treatment with semaglutide could contribute to the clinical outcomes of patients with psoriasis associated with comorbidities such as obesity and T2DM.

## Figures and Tables

**Table 1:** Demographic and clinical data of the studied patients.

Participants	Semaglutide N (%)	Control N (%)	*p*
Sex			
male	12 (80)	13 (81.25)	1
female	3 (20)	3 (18.75)	
Alcohol consumption			
Yes	8 (61.5)	10 (66.7)	1
No	5 (38.5)	5 (33.3)	
Smoking status			
Active smoker	2 (15.4)	5 (33.33)	0.45
Ex-smoker	7 (53.8)	5 (33.33)	
Non-smoker	4 (30.8)	5 (33.33)	
Heart attack			
Yes	1 (7.7)	0	0.46
No	12 (92.3)	15 (100)	
Stroke			
Yes	0	0	1
No	13 (100)	15 (100)	
Age			
mean ± SD	58.6 ± 8.04	57.4 ± 13.02	0.7
BMI (F0)			
mean ± SD	35.04 ± 5.9	36.2 ± 7.7	0.6
PASI score values (F0)			
mean ± SD	20.75 ± 9.72	20.36 ± 8.72	0.9
DLQI score values			
mean ± SD	13 ± 6.5	10.1 ± 4.1	0.14
Glucose (mmol/L) (F0)	7.3 (2.75)	5.95 (3.65)	0.22
Cholesterol (mmol/L) (F0)	5.15 ± 1.07	5.06 ± 1.07	0.81
HgbA1C (%) (F0)	7.3 (0.35)	4.95 (1.2)	0.7

Data are expressed as frequency and mean ± SD or median (interquartile range: IQR). HgbA1c: glycosylated hemoglobin A1c. BMI: body mass index. F0: beginning of study.

**Table 2:** Lipid status, glucoregulation, uric acid and homocysteine at baseline and after 12 weeks.

Semaglutide				Control		
Parameters	F0	F1	*p*	F0	F1	*p*
Lipids						
Cholesterol (mmol/L)	5.2 ± 1.1	4.6 ± 1.2	0.2	5.1 ± 1.1	5.1 ± 1.2	0.5
Triglycerides (mmol/L)	1.8 (0.9)	1.5 (1.5)	0.3	1.6 ± 0.7	1.7 ± 1	0.4
HDL (mmol/L)	1.1 ± 0.2	1.07 ± 0.3	0.4	1.2 ± 0.3	1.2 ± 0.4	0.9
LDL (mmol/L)	3.6 ± 1	2.8 ± 0.9	0.03 *	3.6 ± 1.2	3.4 ± 1	0.2
Glucoregulation						
Glucose (mmol/L)	7.0 (3.2)	6.3 (1.4)	0.3	6.4 (3.7)	6.1 (1.1)	0.8
HbA1C (%)	7.3 (0.2)	6.1 (0.8)	0.02 *	7.2 (0.6)	6.3 (1.7)	0.001 **
Insulin (µIU/mL)	11.4 ± 7.5	10.8 ± 6.7	0.7	11 (20.4)	10.8 (8.7)	0.2
Uric acid (µIU/mL)	361.2 ± 97.9	358.6± 76.6	0.9	382.9 ± 83.2	399.2 ± 100.5	0.4
Homocysteine	9.44 ± 2.21	10.91 ± 2.84	0.15	10.07 ± 2.27	12.32 ± 5.09	0.08

Data are expressed as mean ± SD or median (interquartile range: IQR). * statistical significance, *p* ≤ 0.05; ** statistical significance, *p* < 0.001. F0: beginning of study; F1: end of study; HgbA1c: glycosylated hemoglobin A1c; HDL-C: high-density lipoprotein cholesterol; LDL-C: low-density lipoprotein cholesterol.

**Table 3:** Inflammatory parameters at baseline and after 12 weeks of treatment.

Semaglutide				Control		
Parameters	F0	F1	*p*	F0	F1	*p*
Inflammatory						
CRP (mg/L)	3.8 (3.1)	1.9 (1.4)	0.01 *	9.6 ± 10.7	7.6 ± 8.3	0.5
IL-1β (pg/mL)	0.8 (0.4)	0.6 (1)	0.3	0.5 (0.9)	0.6 (0.6)	0.9
IL-6 (pg/mL)	3.5 (2.3)	2.8 (1.1)	0.05 *	5.6 (12.2)	2.3 (3.6)	0.1
IL-23 (pg/mL)	51.9 ± 30.2	41.2 ± 27.1	0.2	87.5 (59.1)	51.6 (55.2)	0.1

Data are expressed as mean ± SD or median (IQR). * Statistical significance, *p* ≤ 0.05.

**Table 4:** Comparison of CRP and IL-23 values between semaglutide group and control group.

Parameters	F0/Semaglutide	F0/Control	*p*
CRP (mg/L)	3.8 (3.1)	4.8 (9.7)	0.07
IL-23 (pg/mL)	46.08 (47.9)	87.5 (59.1)	0.04 *

Data are expressed as mean ± SD or median (IQR). * Statistical significance, *p* ≤ 0.05.

**Table 5:** Psoriasis severity in patients with semaglutide and control group.

Psoriasis Severity	Semaglutide F0		Semaglutide F1		*p*	Control F0		Control F1		*p*
	N	%	N	%		N	%	N	%	
Mild form of the disease	0	0	6	100	0.01 *	0	0	4	100	0.09
Moderately severe form and severe form of the disease	13	65	7	35		15	57.7	11	42.3	

* statistical significance, *p* ≤ 0.05; F0: beginning of study; F1: end of study.

**Table 6:** DLQI classes in patients with semaglutide and control group.

DLQI Classes	Semaglutide F0		Semaglutide F1		*p*	Control F0		Control F1		*p*
	N	%	N	%		N	%	N	%	
No effect	0	0	2	100	0.04 *	0	0	2	100	0.7
Weak effect	2	20	8	80		2	50	2	50	
Moderate effect	2	50	2	50		6	50	6	50	
Strong effect	9	90	1	10		7	58.3	5	41.7	

DLQI: Dermatology Life Quality Index. * Statistical significance, *p* ≤ 0.05; F0: beginning of study; F1: end of study.

## Data Availability

Data can be provided upon reasonable request by contacting the authors.
